# Human Umbilical Cord Therapy Improves Long-Term Behavioral Outcomes Following Neonatal Hypoxic Ischemic Brain Injury

**DOI:** 10.3389/fphys.2019.00283

**Published:** 2019-03-22

**Authors:** Tayla R. Penny, Amy E. Sutherland, Jamie G. Mihelakis, Madison C. B. Paton, Yen Pham, Joohyung Lee, Nicole M. Jones, Graham Jenkin, Michael C. Fahey, Suzanne L. Miller, Courtney A. McDonald

**Affiliations:** ^1^The Ritchie Centre, Hudson Institute of Medical Research, Clayton, VIC, Australia; ^2^Department of Obstetrics and Gynaecology, Monash University, Clayton, VIC, Australia; ^3^Centre for Endocrinology and Metabolism, Hudson Institute of Medical Research, Clayton, VIC, Australia; ^4^Department of Pharmacology, School of Medical Sciences, University of New South Wales, Sydney, NSW, Australia; ^5^Department of Paediatrics, Monash University, Clayton, VIC, Australia

**Keywords:** umbilical cord blood, behavior, hypoxia ischemia, hypoxic ischemic encephalopathy, cerebral palsy, stem cells

## Abstract

**Background:** Hypoxic ischemic (HI) insult in term babies at labor or birth can cause long-term neurodevelopmental disorders, including cerebral palsy (CP). The current standard treatment for term infants with hypoxic ischemic encephalopathy (HIE) is hypothermia. Because hypothermia is only partially effective, novel therapies are required to improve outcomes further. Human umbilical cord blood cells (UCB) are a rich source of stem and progenitor cells making them a potential treatment for neonatal HI brain injury. Recent clinical trials have shown that UCB therapy is a safe and efficacious treatment for confirmed cerebral palsy. In this study, we assessed whether early administration of UCB to the neonate could improve long-term behavioral outcomes and promote brain repair following neonatal HI brain injury.

**Methods:** HI brain injury was induced in postnatal day (PND) 7 rat pups via permanent ligation of the left carotid artery, followed by a 90 min hypoxic challenge. UCB was administered intraperitoneally on PND 8. Behavioral tests, including negative geotaxis, forelimb preference and open field test, were performed on PND 14, 30, and 50, following brains were collected for assessment of neuropathology.

**Results:** Neonatal HI resulted in decreased brain weight, cerebral tissue loss and apoptosis in the somatosensory cortex, as well as compromised behavioral outcomes. UCB administration following HI improved short and long-term behavioral outcomes but did not reduce long-term histological evidence of brain injury compared to HI alone. In addition, UCB following HI increased microglia activation in the somatosensory cortex compared to HI alone.

**Conclusion:** Administration of a single dose of UCB cells 24 h after HI injury improves behavior, however, a single dose of cells does not modulate pathological evidence of long-term brain injury.

## Introduction

Neonatal brain injury underlies long-term neurological deficits, including cerebral palsy (CP) and other cognitive and behavioral deficits that may become apparent months to years after birth (Vannucci, 2000; Ferrari et al., 2010). A significant cause of neonatal brain injury is severe and/or prolonged hypoxia-ischemia (HI) in utero, at labor or at birth, resulting in a lack of oxygen supply to the brain (Vannucci, 2000). Resulting brain injury is termed hypoxic-ischemic encephalopathy (HIE) which can be diagnosed through routine testing through use of Apgar and Sarnat scores, and MRI, however, the latter is usually performed outside of the early intervention critical treatment window. Because of this, some infants are not diagnosed within the early stages of HIE, thus early intervention therapies cannot be administered (Aridas et al., 2014; Ahearne et al., 2016). Of all neonates with HIE, 10–15% will die shortly after birth, 10–15% will develop cerebral palsy, and 40% will develop other disabilities, such as autism, epilepsy and impairment of cognition, motor abilities and behavioral problems (Cerio et al., 2013). Currently, the only treatment for HIE is therapeutic hypothermia which is effective but also restricted in scope (Cerio et al., 2013) in that treatment needs to commence within 6 h of birth (Chiang et al., 2017), is best applied in well-equipped tertiary hospitals, and is not a suitable treatment for babies born preterm (Bennet et al., 2012). These limitations indicate the need for the development of novel treatment/s, that could be used in conjunction with hypothermia or as a standalone treatment if required. The motor and behavioral impairments associated with HIE may not be diagnosed until many months after birth. For example, cerebral palsy may not be confirmed until 12–24 months of age, when a child is not reaching developmental milestones (Rosenbaum, 2003). It is therefore critical to assess long-term structural and functional outcomes in animal models of early intervention therapies for neonatal HI, with the aim of implementing a therapy that is beneficial for improving long-term neurodevelopmental outcomes.

Human umbilical cord blood cells (UCB) are an abundant source of naïve stem cells that can be easily harvested and feasibly be delivered to newborns within the first day of life, or be banked for later use (Cotten et al., 2014). UCB contains a large population of stem and progenitor cells, including endothelial progenitor cells and haematopoietic stem cells, as well as immunosuppressive cells, such as regulatory T cells and monocyte derived suppressor cells (Torelli et al., 2012; Jaing, 2014). UCB can be administered with little risk of rejection, even when used allogeneically (Maeda et al., 2005). This is partly due to a reduced presence of HLA antigens and, as such, there is a low risk of graft-versus-host disease, when compared to adult-derived cells, such as those derived from bone marrow (Knutsen and Wall, 2000). UCB is already an established treatment option for many conditions and is now under investigation for early intervention (within the first week after injury) in pre-clinical models of neurological injury (Aridas et al., 2016; Li et al., 2016; McDonald et al., 2018). In addition, there are a number of clinical trials underway that are investigating UCB therapy in children and adolescents with established cases of CP, treating months to years after the initial injury. Kang et al. (2015) demonstrated that a high dose of 20 million cells per kg administered to patients between 6 and 20 years old, results in improved motor functions and muscle strength within 6 months of treatment. Min et al. (2013) also showed that patients aged between 10 months and 10 years of age, treated with 30 million cells/kg also showed an improvement in gross motor function and cognitive scores. Data from clinical trials is promising, however, it is still unclear what long-term effects these cells have on behavioral and neuropathological outcomes, particularly following early intervention, when cells are delivered soon after injury.

This study aimed to assess the therapeutic use of human UCB for the treatment of HI brain injury in term-equivalent newborn rats, focused in particular on the analysis of long-term behavioral outcomes, as well as neuropathological outcomes. We hypothesized that a single dose of UCB at 24 h post neonatal HI insult would improve behavioral outcomes, as well as decrease neuronal damage and inflammation in the brain.

## Materials and Methods

### Ethics Statement

All experiments in this project were performed with human ethics approval from Monash Health Human Ethics Committee (12387B) and Animal ethics approval from Monash Medical Centre Animal Ethics Committee A (MMCA/2015/42). Written informed consent was obtained from all participants prior to collection of UCB. All experiments were performed in accordance with the Australian National Health and Medical Research Council guidelines.

### Isolation, Cryopreservation and Preparation of Cells

Human umbilical cord blood was collected via the umbilical vein of healthy term cesarean section births (>37 weeks), into a collection bag containing anticoagulant (Macopharma). The patients gave written informed consent for the collection and research use of cord blood. On average, ∼125 ml of blood was collected from each patient. Blood was stored at room temperature on a shaker for <72 h before processing. To separate the mononuclear cell population, the blood was evenly separated into falcon tubes, diluted with equal amounts of phosphate buffered saline (PBS; Gibco Life Technologies) and centrifuged for 12 min at 3100 RPM at room temperature (RT), with no brake. The mononuclear cell layer was collected and washed with 20 ml PBS and centrifuged at 800 g at RT for 5 min. 20 ml of red blood cell lysis buffer [155 Mm ammonium chloride (NH4Cl: Sigma-Aldrich), 10 mM potassium bicarbonate (KHCO3: Sigma-Aldrich) and 0.1 mM EDTA (Sigma-Aldrich) dissolved in distilled water] was added to the cell pellet to lyse any remaining red blood cells in the sample. The lysis was stopped after 3 min with 30 ml media [10% fetal bovine serum (FBS), 1% antibiotic, 1% L-glutamine in DMEM/F12; all from Gibco Life Technologies] and centrifuged at 400 *g* at RT for 5 min. The cells were counted using trypan blue exclusion dye (Gibco) with a haemocytometer. Samples were then cryopreserved at an approximate density of 20–50 million cells/ml. UCB cells suspended in media were transferred to 2 ml cryopreservation vials and an equal volume of cryopreservation media [80% FBS (Gibco Life Technologies) containing 20% dimethyl sulfoxide (DMSO; Sigma-Aldrich)] was added dropwise, resulting in a final concentration of 10% DMSO. Vials were placed in a freezing container (Mr. Frosty, Thermo Fisher Scientific) and stored at -80°C overnight. Cells were then moved to liquid nitrogen for long-term storage.

### Animals

#### Animal Ethics, Selection and Welfare

Time-mated pregnant Sprague-Dawley (SD) rat dams were sourced from Monash Animal Research Platform and transported to Monash Medical Centre Animal Facility 1 week prior to birth. They were housed in individual boxes in standard housing conditions in rooms with a 12-h light/dark cycle. The dams were allowed to birth naturally and were only disturbed to count the pups on PND 2–3.

A total of 36 rat pups (from 8 dams) were used in this study. Rat pups were randomly assigned to experimental groups, based on surgery times, to control for any neuroprotective effects associated with prolonged isoflurane exposure. 17 animals were exposed to HI (HI, *n* = 11: 3 males and 8 females; HI + UCB, *n* = 6: 3 males and 3 females) and 19 animals received sham surgery with no HI (Sham, *n* = 19: 8 males, 11 females). Sex of the animal was not taken into consideration when performing analysis.

#### Animal Surgery to Induce HI

A permanent unilateral carotid artery ligation was performed on post-natal day (PND) 7, followed by exposure to hypoxia, as previously described (McDonald et al., 2018). The pups were separated from their mother and placed on a 37°C heat pad before and after surgery. The pups were anesthetized by inhalation of 4% isoflurane which was maintained at 1–2% for the duration of the surgery. A small incision was made in the neck, and the left carotid artery was exteriorised before being occluded with an electrocautery device. The wound was sutured closed using 6-0 polypropylene suture and isoflurane was stopped. Bupivacaine was applied to the surgery site for pain management. The pups were returned to their dam for a 1 h recovery period. Following this, the pups that underwent artery ligation were placed in a humidified hypoxic chamber for 90 min at 8% oxygen, balanced with 92% nitrogen, and the chamber was maintained at 35–36°C. Control pups underwent a sham surgery, in which the artery was not ligated. Following surgery, they were returned to their dam for a 1 h recovery period and were then placed on a 37°C heating pad and exposed to normal room air for the same duration as the injury group that underwent hypoxia. Following the hypoxic treatment, the pups were returned to the dam for recovery. For the remainder of the experiment, the pups were health checked daily and weighed 3–4 times a week.

#### Preparation and Administration of UCB Cells

Rat pups received UCB cells 24 h post insult (PND8), via intraperitoneal injection. Cells were thawed rapidly in a 37°C water bath. UCB cells from three different donors were pooled and washed with media to remove DMSO within the freeze media. The cells were counted and viability determined using trypan blue exclusion. Before administration, cells were washed in PBS and resuspended in PBS at a final concentration of 5 million cells/ml. Cells were stored on ice until administration to rat pups.

Pups received 1 million UCB cells (equivalent to 61 × 10^6^ cells/kg) via an intraperitoneal injection in a volume of 200 μl of PBS using a 30-gauge insulin syringe. HI control pups received an injection of 200 μl of PBS only, using a 30-guage insulin syringe.

### Behavioral Testing

#### Negative Geotaxis Analysis

As previously described (McDonald et al., 2018), on PND14 the pups were placed head-down on a 45-degree inclined slope covered with a standard laboratory bench pad. The time taken for them to turn 180 degrees was recorded, and then the time taken to walk ∼15 cm up the slope to cross a line was also recorded. This test was performed 3 times per pup. If the pup took longer than 90 s to complete the test, or if they climbed off the board, it was considered a fail and not included in the analysis. No pups were excluded from analysis in this study.

#### Open Field Test

The open field test was completed on PND 30 and 50 using the Topscan behavioral analysis program to track the movement of the rat. Distance, speed and time moved were recorded for both the inside area and the perimeter of the box. Before testing, the rats were acclimatized to the room for 1–2 h, and the open field boxes were disinfected to remove foreign smells that could influence behavior. For the open field test, the rats were placed in the large open box, approximately 50 cm × 50 cm, for 10 min and allowed to explore the box freely. This test assesses the anxiety behaviors of the rats, as a more anxious rat will spend the time walking the perimeter of the box (Gould et al., 2009).

#### Cylinder Test

On PND 30 and 50, the rats completed the cylinder test to analyze forelimb preference. The rats were placed in a clear glass beaker and video recorded from overhead for 2 min. The cylinder was disinfected between trials to remove foreign smells that could influence behavior. The videos were then analyzed to assess, upon rearing, how often the right and left foot touched the wall of the cylinder. This test assesses if the HI injury has impaired limb use and caused asymmetric motor control.

#### Behavioral Composite Z-Score

All behavioral outcomes were converted into a *Z*-score which was compared to the *Z*-score generated from the sham group. *Z*-score was calculated by subtracting the mean test score of the control group from the individual test score, divided by the standard deviation of the control group.

For the negative geotaxis *z-*score data was transformed from the time to turn and time to cross the line results (Supplementary Figures S1A,B) to generate *z*-scores. For the open field *z*-score data from average time spent in the center of the box (Supplementary Figures S1C,D) was used to generate *z*-scores. For the cylinder test *z*-score data from the percentage of left foot touches (Supplementary Figures S1E,F) was used to generate *z*-scores. These *Z*-scores were summed across the included tests (negative geotaxis analysis, open field test and cylinder test) to give a final cumulative score as an overall indication of behavioral deficits across both motor control and anxiety-like behavior.

### Post-mortem and Brain Processing

On PND 50, following behavioral testing, the rats were culled by lethal inhalation of carbon dioxide, followed by decapitation. Brains were collected and fixed in 10% formalin (Grale Scientific) for 3–4 days, then processed and embedded in paraffin wax. For histological analysis, the embedded brains were coronally sectioned in 6 μm slices.

### Histology

#### Gross Brain Morphology

Gross brain morphology and tissue area were assessed with Haematoxylin and Eosin (H&E, Amber Scientific, Australia). For each animal, triplicate slides over two regions approximately 0.2 and -3.3 mm Bregma were examined and data averaged across groups. Images were acquired by Aperio digital scanning (Leica Biosystems, Germany) and the volume of the left (ipsilateral to the injury) and right (contralateral to the injury) hemisphere were measured using Aperio ImageScope (Leica Biosystems, Germany). For percentage tissue loss, the difference in volume between the contralateral and ipsilateral hemispheres over the contralateral hemisphere volume was calculated, using the following formula [(volume of contralateral-volume of ipsilateral)/volume of contralateral], as previously described (Teo et al., 2017).

#### Immunohistochemistry

Microglia were identified using ionized calcium-binding adapter molecule 1 (Iba-1; 1:1000, Wako Pure Chemical Industries, Ltd., Osaka, Japan), apoptotic cell death was assessed using activated caspase 3 (Cas3; 1:800, R&D Systems, Minneapolis, MN, United States), myelin was assessed using Myelin Basic Protein (MBP; 1:250, Merck, Darmstadt, Germany), neuronal cell counts were assessed using NeuN (1:1000, Millipore, Burlington, MA, United States) and astrocytes were assessed using glial fibrillary acidic protein (GFAP; 1:400, Sigma-Aldrich, Castle Hill, NSW, Australia).

Briefly, brain sections were dewaxed in alcohol (xylene and ethanol), followed by antigen retrieval in heated citric acid buffer. Endogenous peroxidases were blocked by incubating sections with hydrogen peroxide in 50% methanol/dH_2_O. The sections were blocked with serum [Iba1 and MBP: 10% normal goat serum (NGS); GFAP: 5% NGS; Cas3: 5% NGS + 2% Bovine serum albumin (BSA); NeuN: 5% NGS + 1% BSA]. Slides were incubated overnight at 4°C in the specific primary antibody. Sections were exposed to a secondary antibody for 1 h (Iba1 and Cas3: Goat anti-Rabbit IgG, 1:200; Vector Laboratories, Burlingame, CA, United States; MBP: Goat anti-Rat IgG, 1:200; Vector Laboratories, Burlingame, CA, United States; GFAP and NeuN: Goat anti- Mouse IgG, 1:200; Vector Laboratories, Burlingame, CA, United States). Staining was visualized using 3,3-diaminobenzidine (MP Biomedicals, Santa Ana, CA, United States).

Coronal brain sections were imaged using bright field microscopy on the Olympus BX-41 microscope (Olympus, Tokyo, Japan). For immunohistochemical analysis, images were acquired at 400×, with three random non-overlapping fields of view analyzed per region, on two non-adjacent duplicate slides per brain, averaged for each animal. The regions of interest examined included the somatosensory cortex (0.2 mm bregma) and the motor cortex (+3 mm bregma). Cell counts (Iba-1, NeuN, Caspase-3) and densitometry (MBP, GFAP) were preformed using Image J (NIH, Bethesda, MD, United States). Quantification of microglia cell types was achieved by classing the microglia as either resting (ramified, with branching projections protruding from the cell body) or active (amoeboid, no projections seen, cell body is round) (Kettenmann et al., 2011). Aggregates of microglia were observed as dense patches of microglia cell bodies and branching projections. All assessments were conducted on coded slides and images, with the examiner blinded to the experimental groups.

### Statistical Analysis

Statistical analysis was performed using GraphPad Prism 7.0 (GraphPad Software, San Diego, CA, United States). Statistical significance was set at a *P*-value of <0.05, and all data was presented as the mean the standard error of the mean (SEM). Parametric data was analyzed using a one-way ANOVA with Tukey’s *post hoc* analysis. Non-parametric data was analyzed using a Kruskal–Wallis test with Dunn’s multiple comparisons *post hoc* analysis.

## Results

### The Effect of HI Injury and UCB Administration on Behavioral Outcomes

**FIGURE 1:**
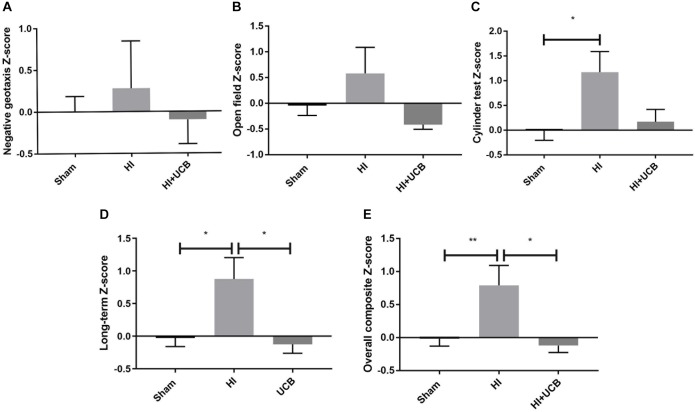
Behavior is impaired by HI injury and is significantly restored following UCB administration. **(A)** *Z*-score of the negative geotaxis analysis at PND 14. **(B)** *Z*-score of overall open field test score from PND 30 and 50. **(C)** *Z*-score of overall cylinder test score from PND 30 and 50. **(D)** A combination of PND 30 and 50 data from the open field test and cylinder test. **(E)** Overall Composite *Z*-score combining data from the negative geotaxis analysis, cylinder test and open field test, from PND 14, 30, and 50 (Data expressed as mean ± SEM, *n* = 6–19 pups per group, ^∗^*P* < 0.05, ^∗∗^*P* < 0.01).

Behavioral test scores from negative geotaxis analysis, open field test and cylinder test, were combined to form a *Z*-score such that overall effects of HI and UCB treatment could be compared relative to sham. Non-transformed behavioral data is included in Supplementary Figure S1. Regarding negative geotaxis analysis, which evaluates both motor strength and the vestibular reflex, the HI group was the only cohort to show a positive *z*-score compared to sham, where a positive score is indicative of worse outcomes, but overall there were no significant differences (Figure 1A). For the open field test, which assesses anxiety/exploratory behaviors, the same group relationships were observed, but also with no significant differences (Figure 1B). The cylinder test assesses forelimb asymmetry, and a significant increase in the *Z*-score in the HI group (*P* < 0.05, Figure 1C) was observed when compared to sham, where the UCB treated group showed no difference to sham (Figure 1C).

In order to ascertain the long term and overall behavioral burden, a long-term composite behavior score was calculated by combining data from D30 and D50 behavioral tests (Figure 1D) and an overall behavioral burden score was calculated by combining each of the individual test *Z*-scores for all behavioral tests (Figure 1E). This overall behavioral burden score incorporated motor strength, anxiety/exploratory behaviors and limb asymmetry. For both the long-term composite *z*-score and the overall behavioral burden, we observed a significant impairment following HI injury when compared to sham (*P* < 0.05 Figure 1D; *P* < 0.01 Figure 1E, respectively). Administration of UCB significantly reduced long-term outcomes (*P* < 0.05, Figure 1D) and the overall behavioral burden (*P* < 0.05, Figure 1E) compared to HI.

### Effect of HI Injury and UCB Administration on Tissue Loss and Apoptotic Cell Death

**FIGURE 2:**
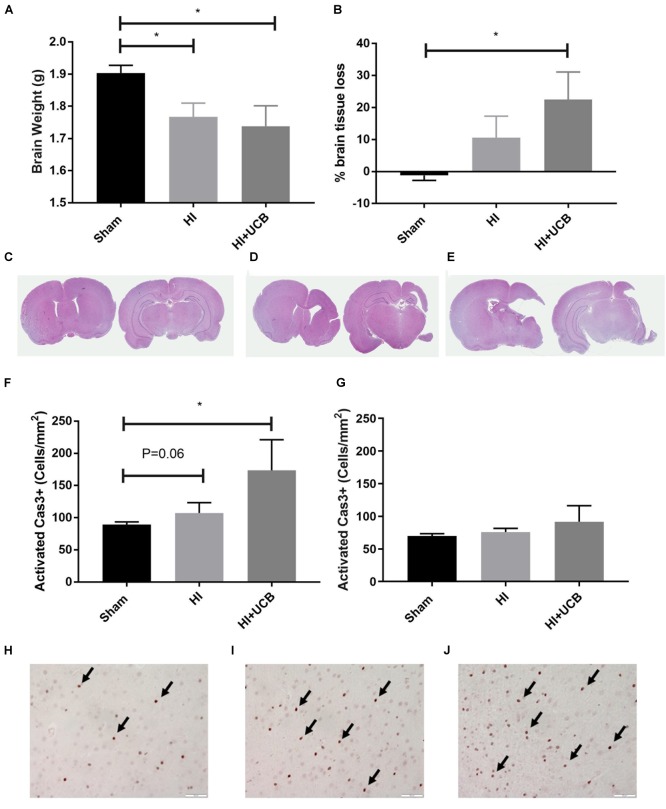
HI injury causes tissue loss and cell death which is exacerbated by UCB administration. **(A)** Brain weights. **(B)** Percentage of left hemisphere volume of the total brain. Representative images of haematoxylin and eosin stained brain sections used for volumetric analysis for sham **(C)**, HI **(D)**, and UCB **(E)** groups. **(F)** Number of activated Caspase 3 positive cells in the somatosensory cortex of the brain. **(G)** Number of activated Caspase 3 positive cells in the motor cortex of the brain. Representative images of Cas3 positive cells (indicated by black arrows) in the somatosensory cortex of the brain in sham **(H)**, HI **(I)**, and UCB **(J)** animals (Data expressed as mean ± SEM, *n* = 6–19 pups per group, ^∗^*P* < 0.05, Scale bar = 50 μm).

The HI brain injury and UCB treatment had no effect on body weight over the course of the experiment, compared to sham (data not shown). At PND 50 (43 days post HI) a significant decrease in brain weight in both the HI and UCB groups (*P* < 0.05; Figure 2A), was observed when compared to the sham group. This brain injury was further confirmed by the substantial loss of brain tissue in the HI and UCB group, with significant tissue loss in the left hemisphere of the UCB group (*P* < 0.05; Figure 2B,D,E), compared to sham (Figure 2C). In addition to these morphological changes, we also saw a trend toward an increase in apoptosis in the somatosensory cortex in the HI (*P* = 0.06, Figure 2F,I) and a significant increase in the UCB group (*P* < 0.05; Figure 2F,J) compared to sham (Figure 2H). There was no significant change in apoptosis between groups in the motor cortex (Figure 2G). There was no significant change in the number of neurons, seen by NeuN immunohistochemistry, and the white matter density between groups in the somatosensory cortex and motor cortex, seen by MBP immunohistochemistry (data not shown).

### Inflammatory Response Following HI Injury and UCB Cell Treatment

**FIGURE 3:**
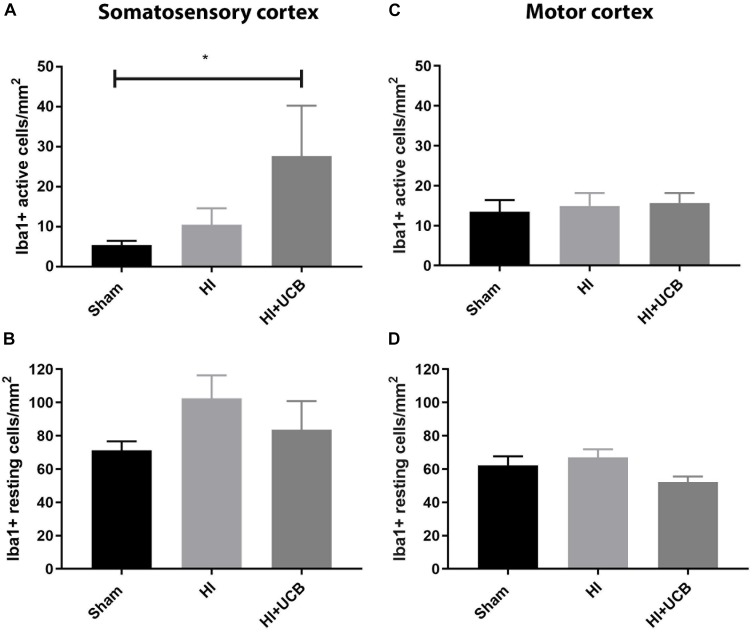
Umbilical cord blood cells delivery increases microglia activation in the somatosensory cortex. **(A)** Number of activated Iba1 positive cells in the somatosensory cortex (SSC) in the brain. **(B)** Number of resting Iba1 positive cells in the SSC. **(C)** Number of activated Iba1 positive cells in the motor cortex (MC) of the brain. **(D)** Number of resting Iba1 positive cells in the MC (Data expressed as mean ± SEM, *n* = 6–19 pups per group, ^∗^*P* < 0.05).

The inflammatory response following HI injury involves activation and proliferation of several immune cell types, including microglia (Liu and McCullough, 2013) and astrocytes and while this was thought to occur acutely, there is emerging evidence that the tertiary phase of inflammation plays a role in long-term outcomes (Fleiss and Gressens, 2012). Our results show that administration of UCB cells resulted in a significant increase in activated microglia (Iba1-positive staining) in the somatosensory cortex when compared to sham (*P* < 0.05; Figure 3A) and this increase was not seen in the HI group. There was no difference observed in resting microglia in the somatosensory cortex between any groups (Figure 3B). When looking at the motor cortex, there was no significant difference in the number of activated or resting microglia in this area (Figure 3C,D). In addition, there was no significant difference in the number of microglia aggregates between groups in both the somatosensory and motor cortex (Data not shown).

**FIGURE 4:**
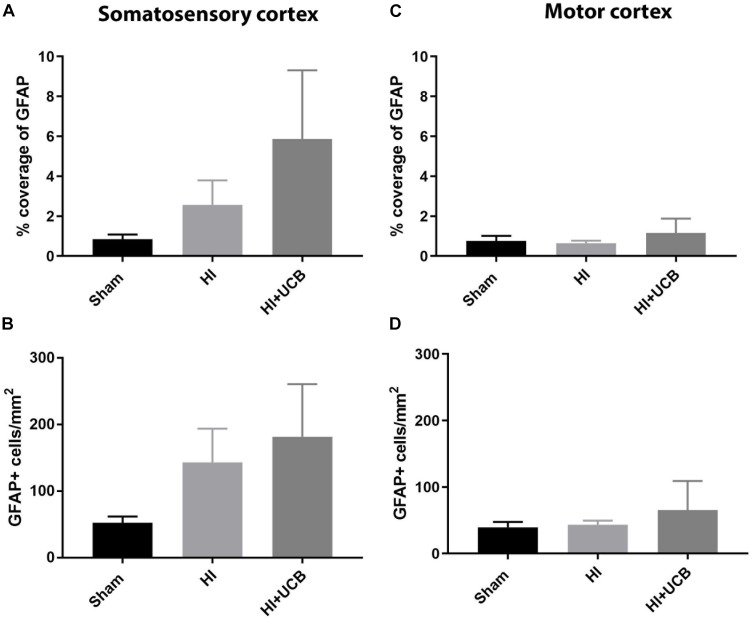
Effects of HI injury and UCB administration on astrogliosis in the somatosensory and motor cortex. **(A)** Percentage coverage of GFAP in the somatosensory cortex (SSC) in the brain. **(B)** Number of GFAP positive cells in the SSC. **(C)** Percentage coverage of GFAP in the motor cortex (MC) of the brain. **(D)** Number of GFAP positive cells in the MC (Data expressed as mean ± SEM, *n* = 6–19 pups per group).

Following HI injury and UCB treatment, we observed no significant changes in astrogliosis, as indicated by GFAP staining, for either astrocyte cell number or percentage coverage in both the somatosensory cortex (Figure 4A,B, respectively) and motor cortex (Figure 4C,D, respectively).

## Discussion

In this study, we investigated the long-term effects of UCB administration for term HI brain injury, particularly regarding behavior, and neuropathology. UCB therapy for HIE and CP has been explored in many preclinical studies and clinical trials and has been shown to improve short-term motor function, muscle strength and cognitive abilities (Kang et al., 2015; Romanov et al., 2015; Jensen and Hamelmann, 2016). In addition, we have previously shown that UCB reduces neuroinflammation and short-term behavioral outcomes in animal models (Aridas et al., 2016; McDonald et al., 2018); however, the long-term effects of UCB administration, particularly regarding behavioral outcomes, have not been widely investigated. It is important that these aspects are investigated to ensure that UCB is not only an effective treatment, but that it is also a sustainable treatment that will show benefits throughout the patient’s life.

This study shows for the first time that UCB cell treatment significantly improves behavioral outcomes in the long-term. However, contrary to these positive behavioral effects, a single dose of UCB cells 24 h after a HI insult did not mitigate neuropathology. We provide evidence that UCB cell treatment may even exacerbate injury in particular regions, such as the somatosensory cortex where we observed increased tissue loss, apoptosis and microglial activation compared to the sham group. Nevertheless, this potential exacerbation of injury appeared to be region specific and importantly, was not significantly increased compared to the HI group.

CP is an umbrella term referring to a group of permanent movement disorders with associated deficits in cognition, perception and behavior (Novak et al., 2012). These commonly seen impairments highlight the need to investigate a therapy that will not only target injurious mechanisms, such as inflammation, and repair brain injury but will also improve behavioral outcomes. In this study, we standardized and transformed individual behavior scores into *Z*-scores, which were then combined to create an overall behavioral burden score. This composite scoring method is standard practice in neurological clinical trials to evaluate changes in motor ability over time in patients with multiple sclerosis (Fischer et al., 1999) and Parkinson’s disease (Stocchi et al., 2018). Our previous study analyzed short-term behavioral deficits following HI and a single dose of UCB cells and found that 7 days after injury, UCB cells resolved impairments seen in the negative geotaxis test, indicating improved muscle strength and coordination (McDonald et al., 2018). In this study, we investigated the overall effects on behavior by examining the functional outcomes at 7, 23, and 43 days post injury and also the long-term subset at 23 and 43 days post injury. These time points were chosen as they correlate to the toddler stage (Colver et al., 2014), pre-pubescent and pubescent stages, respectively, in human development (Bolton et al., 2015). It is important that we look at these timepoints as motor and behavioral deficits associated with CP are often undetected until 12–24 months of age (Rosenbaum, 2003), but persist throughout an individual’s life. This study showed that forelimb asymmetry was present after HI injury, which resulted in preference of the left forelimb. UCB administration reduced this asymmetry, where treated animals were mostly supporting on both forelimbs. In addition, the increased composite *Z*-score for the HI group reveals poor behavioral outcomes and highlights deficits in vestibular reflex, motor strength, coordination, anxiety behaviors, exploratory behaviors and symmetric limb use. UCB administration significantly reversed these behavioral deficits compared to the HI group, thus returning the *Z*-score to sham level. These results indicate that early administration of a single dose of UCB cells effectively treats motor and behavioral deficits following HI brain injury. These results are consistent with current clinical trial data to show that administration of UCB cells to children with established CP can improve motor function, strength and cognition (Kang et al., 2015; Jensen and Hamelmann, 2016). Given the lack of correlation between our behavioral outcomes and neuropathology, it may be worth considering other neurological tests that may be more relevant in future studies. These could include adhesive removal test and novel object recognition (Patel et al., 2015), however, it has been shown that spontaneous recovery in tests such as adhesive removal test is possible, while deficits in novel object recognition appear to be sustained long-term (Liguz-Lecznar et al., 2014).

Despite these beneficial effects of UCB in restoring motor and behavioral deficits, this improvement was not associated with a decrease in brain injury. We showed that HI significantly reduced brain weight and led to tissue loss and a trend toward increased apoptosis in the somatosensory cortex. Treatment with UCB did not restore brain weight or reduce tissue loss or apoptosis and may even exacerbate injury in specific regions, however, this was not significant compared to HI. Our previous study showed that, when UCB treatment was assessed 6 days after administration, UCB cells recovered tissue loss and reduced apoptosis (McDonald et al., 2018). Together our results indicate a single dose of UCB cells may be able to mediate injury early on, but this effect may not be sustained and as such increased doses and timing of administration needs to be examined. The development of HIE and cell degeneration after a severe asphyxic insult at birth is known to progress over time and is traditionally divided into different phases; the *primary*, *latent*, *secondary*, and *tertiary*. The primary phase of injury, which hypothermia targets, describes the period at which the injury is sustained (Gunn and Thoresen, 2006) and is shortly followed by the latent phase at 6–15 h after injury (Gunn and Thoresen, 2006). The secondary phase of injury is the point at which the majority of the damage is thought to occur, due to the acute upregulation of inflammatory pathways, oedema formation, cell death and breakdown of the blood–brain barrier (Gunn and Thoresen, 2006; Kaur et al., 2006; Thornton et al., 2012). This phase has been shown to last as little as 6 h and as long as 3 days in humans, and is a critical window of opportunity for treatment of HIE (Drury et al., 2010). This is the phase that we chose to target in this study by administering UCB cells at 24 h. The tertiary phase of injury can last weeks to years following the initial insult (Fleiss and Gressens, 2012), and is characterized by persisting brain lactic acidosis, epigenetic changes, persistent inflammation and aberrant gliosis, which may inhibit remyelination and regrowth of axons (Thornton et al., 2012; Hassell et al., 2015). One of the key contributing processes to injury during this phase is persistent inflammation. This inflammation has been shown to impede proliferation of cells in the brain, as well as synaptogenesis (Hassell et al., 2015). These prolonged injurious mechanisms could indicate that only targeting the secondary phase of injury with UCB cells is not sufficient for long term repair of the injured brain, and additional doses of UCB cells during the tertiary phase may be required for long-term neuropathological improvements.

Neuroinflammation plays a crucial role in mediating brain injury following a HI event, and is known to persist for many years after the initial injury (Fleiss and Gressens, 2012; Liu and McCullough, 2013). Despite the importance of microglia in the inflammatory response, there have been very few long-term studies looking at long-term microglial activation in any animal models or human cases of HIE (Fleiss and Gressens, 2012), thus we do not know how the microglia act at these long-term time points, or what their morphology might be. In this study, we showed that 43 days after injury, microglial activation remained increased compared to sham in the somatosensory cortex following UCB administration. This increase was not seen in the motor cortex, and there were no differences in resting microglia in either region. The presence of activated microglia for an extended period after injury indicates that there is either ongoing inflammation or repair; thus, microglia may be either in their classically activated M1 state, or in their alternatively activated M2 state. M1 microglia are pro-inflammatory and are activated in response to brain injury or inflammation (Lull and Block, 2010; Cherry et al., 2014). They are classified by their release of pro-inflammatory cytokines, reactive oxygen species and reactive nitrogen species (MacMicking et al., 1997; Cherry et al., 2014). M2 microglia are anti-inflammatory microglia, and express receptors that work to downregulate the inflammatory response and initiate tissue repair (Varin and Gordon, 2009). We hypothesize that the increase in activated microglia in the somatosensory cortex following UCB administration may be contributed by M2 activated microglia which could be working to activate reparative mechanisms. However, given we also observe increased apoptosis and tissue loss in the same region, this may be incorrect. Further investigation is needed to ascertain the long-term role of microglia following UCB cell treatment.

Our results demonstrate that there was an overall improvement in long-term behavioral outcomes following UCB administration, but this was not reflected in improvement in brain pathology, however, it also didn’t exacerbate the injury compared to HI. In our study, the infarct was primarily localized to the somatosensory regions of the brain as is common in this model of injury. The damage to the motor cortex region of the brain was less severe, which may reflect improved coordination, motor control and learning abilities (Kawai et al., 2015), that were assessed with our behavioral tests, which could explain the disparity between pathological and behavioral outcomes. Another reason for this difference, may be that a single dose of UCB may have been sufficient to improve neuronal connectivity, which led to improvement in behavior. In a recent human study investigating the potential of UCB cells for Autism (Carpenter et al., 2019), they demonstrated that single infusion of UCB improved white matter connectivity as shown with MRI. In the context of our model, where there is a significant degree of injury, it may be that a single dose can improve behavioral outcomes, possibly through improvements in connectivity, but is not enough to improve neuropathology outcomes. Further optimisation of this UCB cell therapy is needed. In particular, it is important to explore different dosing regimens and test multiple doses of cells in order for this therapy to be appropriate for use in a human clinical cohort. In light of the new evidence for improved connectivity, MRI analysis of connectivity should also be incorporated into these preclinical investigations.

Limitations of this study include the variability that occurs with the HI rodent model. Despite there being clear injury in the HI group, shown by a decrease in brain weight, there was large variation in the size of the infarct between individual pups in both the HI and UCB groups. This variability was also obvious in the UCB group that had large variations for both microglial and astrocyte analysis. This may be overcome by including more animals to examine these indices in future long-term studies. It is understood that cell populations found within UCB are not always consistent between samples with different proportions of stem/progenitor cells. While we did try to reduce the impact of donor variation by pooling cells from multiple UCB donors before administration of UCB, this is an additional limitation that may have added to variation in the UCB treatment group observed in some of our results. In future studies, it is also important to assess adjuvant cell therapies alongside hypothermia, as hypothermia is the current standard of care in cases of term HIE. Studies by Park et al. (2015) have shown that a combination therapy of hypothermia and UCB-derived mesenchymal stem cells was more effective at attenuating injury, as well as improving behavioral function, than either therapy alone.

## Conclusion

In summary, we show that early administration of a single dose of UCB cells reduces long-term behavioral deficits following HI. We also show that, when a single dose of human UCB cells are administered 24 h after injury, UCB cells are unable to reduce indices of neuropathology in all regions of the brain, despite improvements in behavioral outcomes. This study supports the use of UCB as a treatment for neonatal HI brain injury and shows that further investigation into long-term outcomes is needed. Given that a single dose of UCB cells does not prevent tissue injury, it is clear that multiple doses of UCB cells should be considered at different time points, particularly the tertiary phase where ingoing inflammation may still be contributing to injury. By doing this, UCB cells will be able to target both the secondary and tertiary phase of injury, thus maximizing the therapeutic efficacy of UCB cells for neonatal HI brain injury.

## Data Availability

The datasets generated for this study are available on request to the corresponding author.

## Author Contributions

TP designed, performed, and analyzed all experiments and was a major contributor in writing the manuscript. AS, JM, MP, and YP performed UCB isolations and animal experiments. JL, NJ, GJ, MF, and SM designed experiments and interpreted the data. CM developed idea, sought funding for project, designed and performed experiments, and interpreted data. All authors contributed to the writing, editing, and final approval of the manuscript.

## Conflict of Interest Statement

The authors declare that the research was conducted in the absence of any commercial or financial relationships that could be construed as a potential conflict of interest.
