# Supplementary material for: Human Umbilical Cord Therapy Improves Long-Term Behavioral Outcomes Following Neonatal Hypoxic Ischemic Brain Injury
Source: Front Physiol. 2019 Mar 22;10:283. doi: 10.3389/fphys.2019.00283 (PMC6440382; doi:10.3389/fphys.2019.00283)
Supplement: Supplementary file 1 [file Image_1.pdf]

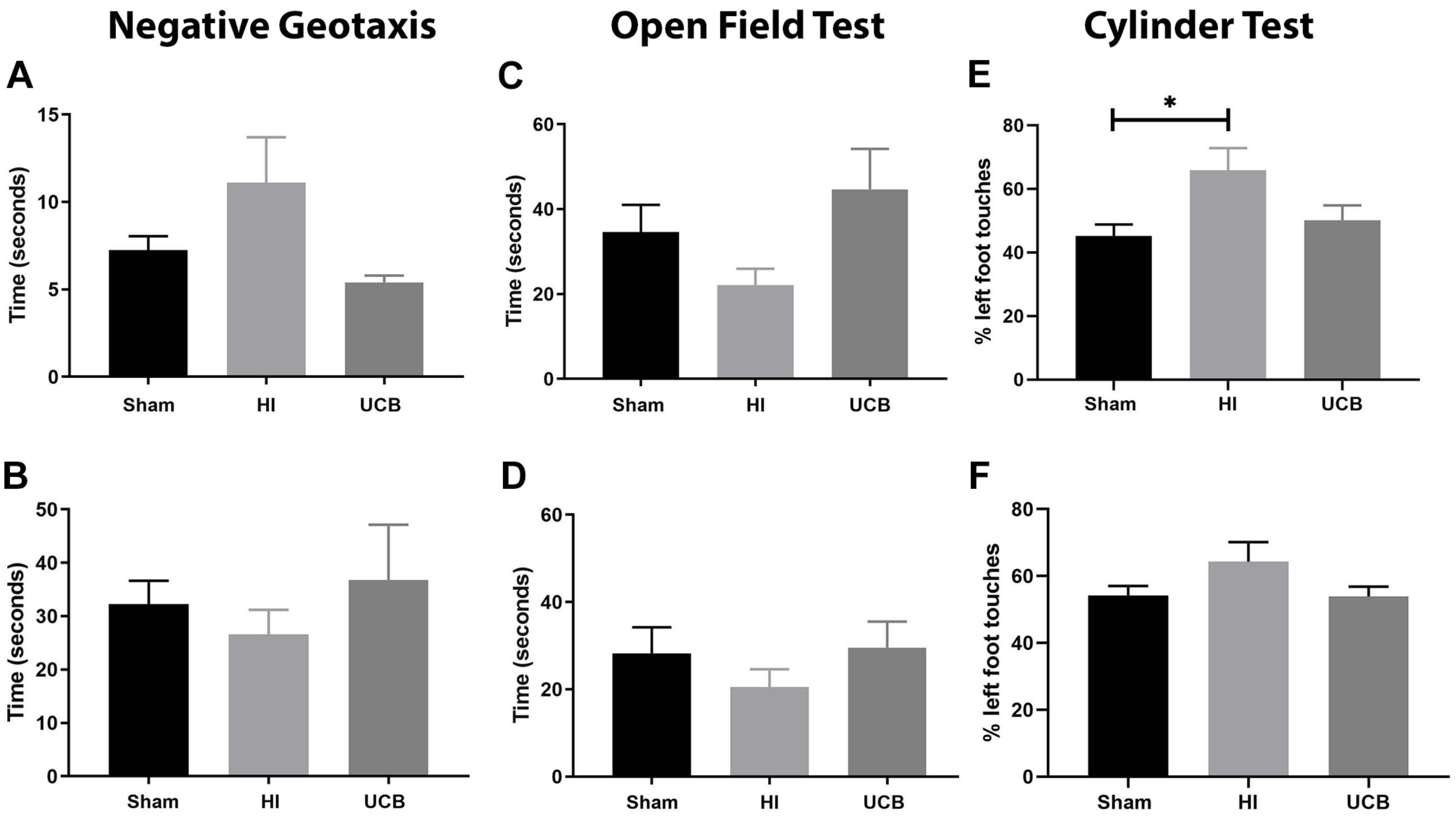

Supplementary Figure 1: Raw behavioural data from individual behavioural tests.
a Time to turn data from negative geotaxis analysis on PND14. **b** Time to cross the line data from negative geotaxis analysis on.PND14. **c** Average time spent in the centre of the box from open field testing on PND30. **d** Average time spent in the centre of the box from open field testing on PND50. **e** Percentage left limb touch from the cylinder test on PND30. **f** Percentage left limb touch from the cylinder test on PND50. (Data expressed as mean ± SEM, n=6-19 pups per group, *P<0.05)
